# Syphilis of the Brain

**Published:** 1882-10

**Authors:** 


SYPHILIS OF THE BRAIN.

The knowledge of syphilis of the nerves and brain has been but lately developed. Virchow speaks first about caseous infiltration of the brain caused by syphilis. Hoyak describes a thrombosis of the arter. fossæ Sylvii with softening of the brain. Esmarch observed cases of mental derangement originating from syphilis. Weber and Wagner showed the presence of nodular growths (syphilomata) on the large vessels of the brain. Heubner's great work on diseased blood-vessels of the brain throws a full light on these pathological phenomena. Prof. Neumann, at Vienna, says that syphilitic degeneration of the central nervous system is discovered by several symptoms. There is intense headache, caused by affection of the dura mater, while dizziness and decrease in the mental functions are effected by impediments in the circulation, *i. e.*, by the diseased vessels which supply the exterior membranes of the brain (Rindenbezirke). The symptoms of a brain affected with syphilis begin suddenly with fainting spells and convulsions, with partial paresis. They do not continue very long, and there are no true paralytic affections; but sometimes there is paresis of the eye or face. From the cerebral symptoms alone it is difficult to make a diagnosis, but there are often affections of the skin, the mucous membranes or the bones. Prof. Neumann had several cases of this kind. The first case was a woman thirty-two years of age, who was delivered of a still-born child two years before, the father of this child being syphilitic. There was a slight erosion on the under lip of the patient, and she had suffered during the past year from epileptic attacks. These attacks had occurred very seldom at first, but afterward they appeared six times in one night. She had made several attempts to escape through the windows. After the use of about 100 grammes of iodide of potassium, all the symptoms disappeared. The second case, a woman twenty-six years of age, had ulcerations on the lower extremities during the last six years. The soles of the feet showed deep scars as a result of psoriasis syphilitica. On the right tibia there were exostoses. There were first fainting spells, with spasms of the muscles of the face; then epileptic attacks, strabismus convergens, and tonic spasms in the lower and upper extremities, with dilated pupils. At the end of four months, the girl was dismissed as cured. The third case showed in the post-mortem examination a softening of the ganglia, caused by obliteration of both exterior fossæ Sylvii. The fourth case, a man thirty-four years of age, had syphilitic ulcerations on the palate, symptoms of ataxy, disturbances of coördination in the right limb, and paræsthesia in the right hypochondrium. He was entirely cured by the use of iodide of potassium. —*Wiener Med. Wochenschr.*

